# Carboxymethyl chitosan-grafted polyvinylpyrrolidone-iodine microspheres for promoting the healing of chronic wounds

**DOI:** 10.1080/21655979.2022.2054911

**Published:** 2022-03-24

**Authors:** Jie Yu, Pei Wang, Mengting Yin, Kaiwen Zhang, Xiansong Wang, Bing Han

**Affiliations:** a Department of Endocrinology, Shanghai Ninth People’s Hospital, Shanghai Jiao Tong University School of Medicine, Shanghai, China; b Department of Thoracic Surgery, Shanghai Key Laboratory of Tissue Engineering, Shanghai Ninth People’s Hospital, Shanghai Jiao Tong University School of Medicine, Shanghai China

**Keywords:** Carboxymethyl chitosan, polyvinylpyrrolidone-iodine, microspheres, chronic wounds, healing

## Abstract

Chronic wounds that fail to heal are the most common complications experienced by diabetic patients, and current treatment remains unsatisfactory, mainly due to the vulnerability of diabetic wounds to bacterial infections. Chitosan derivatives are widely used to treat chronic wounds due to their excellent hydrophilicity, biodegradability, and antimicrobial activity and substantial contribution to tissue regeneration. However, the antimicrobial effect of chitosan is not sufficient due to the complicated pathological mechanism of diabetes mellitus. Here, we prepared carboxymethyl chitosan-grafted polyvinylpyrrolidone-iodine (CMC-g-PVPI) microspheres and used them to treat chronic wounds. Carboxymethyl chitosan (CMC) was used as the skeleton and was grafted with polyvinylpyrrolidone-iodine (PVPI) to form a CMC-g-PVPI complex hydrogel and CMC-g-PVPI microspheres, which formed as a result of the high shearing dispersion of the complex hydrogel. In vivo experiments on diabetic wounds revealed significantly accelerated wound closure in the presence of the microspheres, demonstrating the excellent potential of CMC-g-PVPI to promote skin wound regeneration under diabetic conditions.

## Graphical abstract

**Figure:**
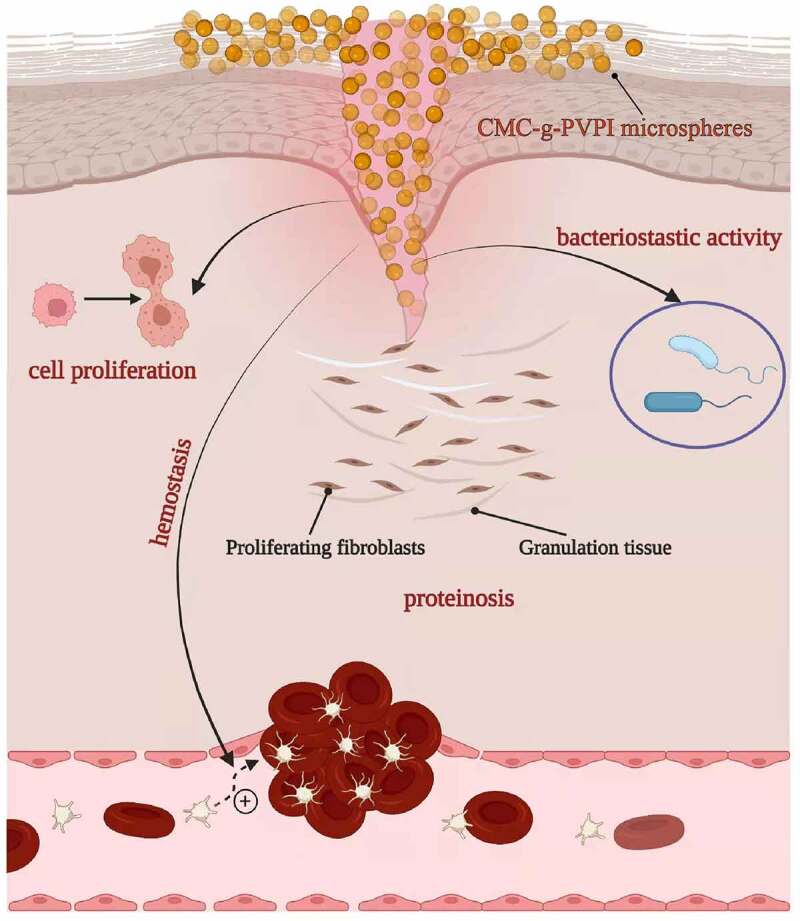

## 1. Introduction

Diabetes is a common global disease that threatens human lives. According to the International Diabetes Federation (IDF) Diabetes Atlas, almost half a billion people have diabetes worldwide, and this number is rapidly increasing [1]. People living with diabetes develop various complications, usually resulting in severe impacts on their health and reducing their quality of life. Diabetic skin wounds are a common but extremely serious complication of diabetes. It is estimated that 15%~25% of diabetic patients will develop diabetic wounds in their lifetime, leading to a high risk of lower extremity amputation and 5-year mortality [2,3]. Due to their high-glucose environment, diabetic wounds exhibit substantial resistance to healing [4]. The failure of diabetic wounds to heal is due to a combination of risk factors, and deficiency of angiogenesis, excessive inflammation and increased susceptibility to infection play significant roles, impeding proper wound healing [^5–7^]. Thus, effective wound treatment focusing on accelerated healing processes and antibacterial properties is crucial for healing diabetic wounds.

In recent decades, the use of natural polymers, including hyaluronic acid, fibrin and chitosan, as scaffolds for wound dressing has attracted intense interest and has great potential for application in diabetic wound healing [^8–10^]. Among the currently available biomaterials, chitosan is one of the most versatile and commercially important natural polymers. It is a cationic polysaccharide mainly derived from the deacetylation of chitin, which can be extracted from the shells of crabs and prawns [11]. Because of its excellent hydrophilicity, biodegradability, biocompatibility, and nontoxicity, chitosan has been widely used in tissue engineering and wound dressing [^12–14^]. Due to its hemostatic properties, chitosan also helps in the wound healing process. The depolymerization of chitosan releases *N*-acetyl-β-D-glucosamine, which induces cell proliferation and collagen deposition, further contributing to tissue regeneration [15]. The unique features of chitosan include antimicrobial activities; however, its antibacterial activity is not sufficient, and chitosan needs to be combined with other antibacterial agents to effectively achieve wound healing [12]. One of the limitations of chitosan is its insolubility at neutral pH [16]. Carboxymethyl chitosan (CMC) is a widely used derivative of chitosan obtained through carboxymethyl modification. It is water soluble and can be degraded by lysozyme [17].

Antibiotics can be classified into bactericidal drugs, which cause cell death, and bacteriostatic drugs, which affect the growth of cells [18]. Among clinical antiseptics, iodophor, also called complexed iodine, has enjoyed widespread use in clinical applications for more than 20 years [19,20]. Polyvinylpyrrolidone-iodine (PVPI), one of the most widely used iodophors, has exhibited an excellent treatment effect in the clinic [21]. In most cases, PVPI behaves as a broad-spectrum antiseptic, showing efficacious bactericidal and virucidal activity [22]. However, despite its less deleterious effects on wounds compared with iodine, PVPI has contributed poorly to the therapeutic wound healing process. Wound repair and antibacterial interventions are often divided into separate steps in clinical treatment. Thus, mutual benefits have emerged when PVPI was combined with chitosan and formed a copolymer with improved antimicrobial performance and wound healing properties.

In the present study, we used CMC as a skeleton and grafted PVPI onto it to form a CMC-g-PVPI complex hydrogel. The CMC-g-PVPI complex hydrogel was synthesized via state-of-the-art methodology [21]. γ-Poly glutamic acid (γ-PGA) is a natural polymer with good biocompatibility, high hydrophilicity and high viscosity [^23–25^]. Herein, we added γ-PGA to the hydrogel to increase the viscosity and hydrophilicity of the product (CMC-g-PVPI-γ-PGA). First, we investigated the healing ability of CMC-g-PVPI-γ-PGA and found that the complex hydrogel did not present a remarkble accelerating effect. Thus, we dispersed the CMC-g-PVPI complex hydrogel into uniform microspheres using high-shear homomixers. The properties of the CMC-g-PVPI microspheres were characterized by scanning electron microscopy (SEM) and Fourier transform infrared (FTIR) spectroscopy. After testing these properties, the CMC-g-PVPI microspheres were applied to a diabetic wound surface to study their wound healing effect.

## 2. Methods

### 2.1. Materials

CMC was purchased from Shanghai Macklin Biochemical Co., Ltd. (China). Azodiisobutyronitrile (AIBN) and methyl methacrylate were purchased from Shanghai Aladdin Biochemical Technology Co., Ltd. (China). Polyvinylpyrrolidone (PVP) was purchased from Sigma–Aldrich (Shanghai) Trading Co. Ltd. (China). γ-PGA (Mw = 1000–15,000) was purchased from Saitaisi Biological Technology Co., Ltd. (China). Iodine, potassium iodide, tert-butanol, acetic acid and sodium dodecyl sulfonate were provided by the laboratory at analytical grade.

### 2.2. Synthesis of CMC-g-PVPI products

CMC-g-PVPI products were synthesized by following a published method with some modifications [21]. First, 50 g CMC was added to 1000 ml water. The solution was stirred at 80°C until the CMC dissolved, and then the solution was filtered through a sand core funnel. Then, 1 g AIBN was added to the filtered solution, and stirring was continued at 80°C for 1 h under nitrogen to initiate graft polymerization. AIBN was used as the initiator and catalyst. Then, 100 g PVP was dissolved in 100 ml water and added to the above solution, followed by 10 ml methyl methacrylate; the mixture was stirred for 30 min, at which point it turned white, and then stirring continued for 4 ~ 6 h. The white solution was concentrated by vacuum distillation to 500 ml, and acetic acid was used to adjust the pH of the solution to 3 ~ 4. Next, 50 g iodine and 40 g potassium iodide were dissolved in 80 ml water. After that, 530 ml *tert*-butanol was added, and the solution was stirred for 1 h. Sodium dodecyl sulfonate (0.25 g) was added to the solution in the second step and stirred until it dissolved.

The white solution was added dropwise into the above 610 ml liquid through a tap funnel. The mixture was then stirred for 2 h to obtain the CMC-g-PVPI complex hydrogel. We dispersed the CMC-g-PVPI complex hydrogel into homogenized microspheres using high-shear homomixers from Shanghai Xin Sha Machinery Co., Ltd. (China), to obtain CMC-g-PVPI microspheres.

Then, 400 µl water was added to 200 µl CMC-g-PVPI complex hydrogel to obtain a mixed 600 µl solution. γ-PGA (350 mg) was dissolved in the solution, and the resulting solution was ultrasonicated for 30 min in an ultrasonic cleaner. It was briefly centrifuged to produce CMC-g-PVPI-γ-PGA.

### 2.3. Characterization of the CMC-g-PVPI microspheres

Samples of CMC-g-PVPI microspheres were sputter-coated with Au and imaged by scanning electron microscopy. The acceleration voltage was 10 kV.

FTIR spectra were obtained with a NEXUS-470 FTIR series spectrometer (Thermo Scientific Nicolet iN10) via the KBr pellet pressing method. KBr pellets and samples were ground at a ratio of 70:1 and mixed uniformly. The instrument was preheated for 20 min before the operation. Software was used to adjust the spectral range to 400–4000 cm^−1^, and 64 scans were performed. First, we scanned the blank background, and then we placed the sample in the instrument and measured the Fourier infrared absorption peak.

### 2.4. Diabetic wound healing test

Animals: Db/db mice were obtained from the Ninth People’s Hospital Animal Center (Shanghai, China) and the experimental protocol was approved by the Animal Care and Experiment Committee of the Ninth People’s Hospital (Ethical approval No.: SH9H-2021-A1009-SB). Fifteen diabetic mice were randomly divided into 3 groups (n = 5). An 8 mm diameter round wound was made on the center of the back of each mouse. Mice in the control group were not treated. The wounds of the mice in the gel group were treated with 200 µl hydrogel. The wounds of the mice in the microsphere group were treated with 10 mg CMC-g-PVPI microspheres. Each mouse was kept isolated in its own cage and supplied with sufficient water and food. The day the wounds were created was defined as Day 0, and photographs of the wounds were taken on Days 0, 3, 5, 7, 9, 12, and 14 to evaluate the healing status. The wound area was calculated using Image-Pro Plus microscope software by applying the following formula:

$$Wound\;area\;\%\;=\;\frac{Actual\;wound\;area}{Original\;wound\;area}$$

### 2.5. Wound histological evaluation

The mice were all sacrificed after 14 days of treatment, and wound samples were collected and fixed in 4% paraformaldehyde overnight. Then, the samples were embedded in paraffin. Each sample was cut into 5 µm thick sections and prepared for histological staining. The formation of granulation tissue was observed by hematoxylin-eosin staining (H&E), and collagen formation was detected through Masson trichrome staining. The tissue sections were incubated with a rabbit monoclonal anti-CD31 antibody (GB113151; Servicebio, 1:300) and an anti-Ki67 antibody (GB111141; Servicebio, 1:500). An HRP-labeled polymer anti-rabbit antibody (GB23303; Servicebio, 1:200) was used as the secondary antibody. Image-Pro Plus 6 software (Media Cybernetics) was used in this study to calculate the CD31 vascularity and the dermis proliferation index.

### 2.6. Data analysis

Statistical analysis was performed using GraphPad Prism Version 8.0.1 (California, USA). Unpaired Student’s t test (and nonparametric tests) were used in this study. Two-tailed analyses were conducted, and P values less than 0.05 (*) and 0.01 (**) were considered significant.

## 3. Results

**Scheme 1.**
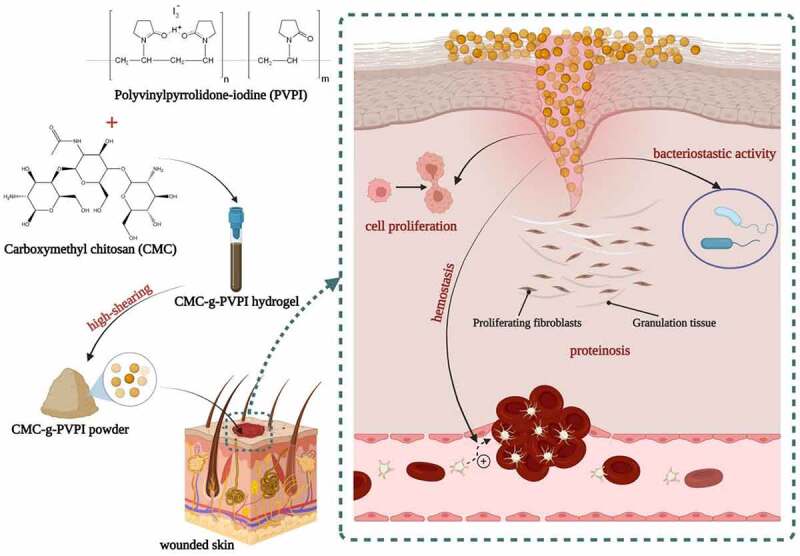
Schematic illustration of diabetic wound healing with the application of CMC-g-PVPI microspheres.

The objective of this study was to fabricate a copolymer with inherent wound healing activities and antibacterial properties to efficiently promote diabetic wound closure. We synthesized two different forms of the CMC-g-PVPI complex: the hydrogel form and the microsphere form. Then, we characterized the complexes and applied them to diabetic wounds to study their healing properties. A schematic representation of this study is shown in Scheme 1.

### 3.1. Preparation and characterization of the CMC-g-PVPI complex hydrogel and CMC-g-PVPI microspheres.

**Figure 1.**
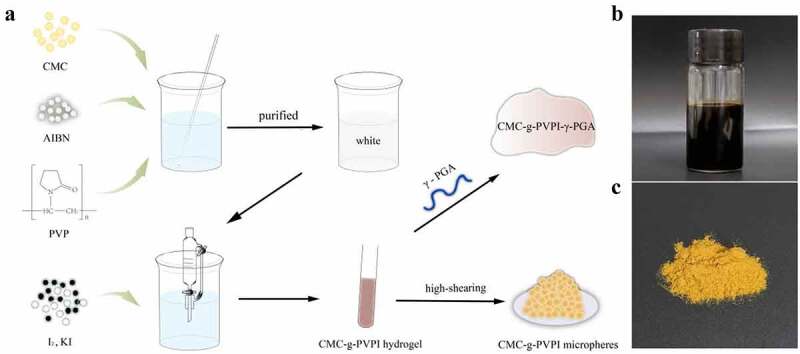
(a) Diagram of the synthesis process of CMC-g-PVPI-γ-PGA and CMC-g-PVPI microspheres. (b) and (c) Appearances of the produced CMC-g-PVPI-γ-PGA and CMC-g-PVPI microspheres.

Figure 1a presents the synthesis process of the powder and hydrogel complexes. The mixed solution of CMC, AIBN and PVP turned white after blending and stirring. AIBN served as the initiator, catalyst, and nitrogen source. The final CMC-g-PVPI microspheres showed a ginger appearance, as shown in Figure 1c, b shows the hydrogel form of the complex production.

**Figure 2.**
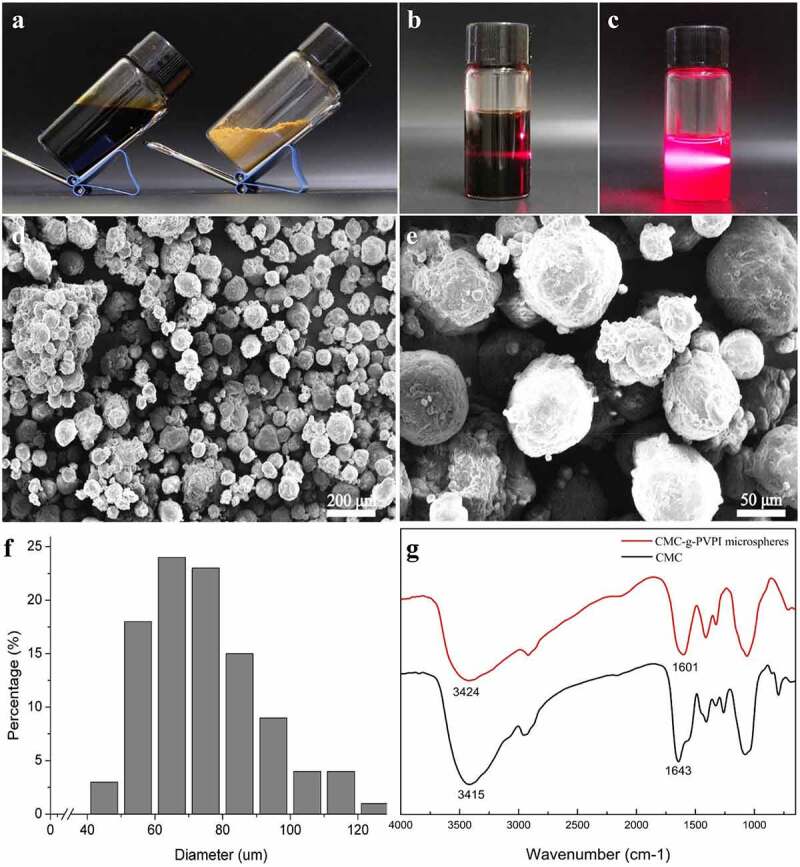
Synthesis, production and characterization of the CMC-g-PVPI complex hydrogel and CMC-g-PVPI microspheres. (a) Photograph of the formed CMC-g-PVPI complex hydrogel and CMC-g-PVPI microspheres. (b-c) Tyndall effect of the CMC-g-PVPI complex hydrogel and CMC-g-PVPI microspheres in deionized water. (d-e) SEM images of CMC-g-PVPI microspheres. (f) Size distribution of CMC-g-PVPI microspheres. (g) FTIR spectra of CMC and CMC-g-PVPI microspheres. (Black) CMC, (red) CMC-g-PVPI microspheres.

Figure 2a shows the final appearance of the CMC-g-PVPI complex hydrogel and CMC-g-PVPI microspheres. The CMC-g-PVPI complex hydrogel appeared brownish red and viscous, and the CMC-g-PVPI microspheres appeared as ginger-colored particles. Figure 2b-c shows the Tyndall effect of the CMC-g-PVPI complex hydrogel and the CMC-g-PVPI microspheres in deionized water. The Tyndall effect arises from the scattering of light by particles in a solution. Under a red laser pointer, the CMC-g-PVPI complex hydrogel and CMC-g-PVPI microsphere solution both manifested visible light paths, illustrating the heterogeneous nature of the two solutions and the uniformly dispersed colloidal particles.

The surface morphology of the CMC-g-PVPI microspheres is presented in Figure 2d-e. The SEM images of the CMC-g-PVPI microspheres revealed that the iodine microspheres had a uniform size and were uniformly distributed in the CMC chain molecules. The copolymer macromolecules showed an average diameter of 74.89 ± 17.19 µm (Figure 2f). PVP has a polar group and an amide group, making it a soluble electron acceptor, and can thus induce the combination of molecules such as iodine with the complexes [21].

We used FITR to characterize the CMC-g-PVPI microspheres. Figure 2g shows the spectrum of the CMC powder, with an obvious absorption peak at 3415 cm^−1^, which was attributed to strong stretching vibrations of the -OH and N-H bonds. In the spectrum of the CMC-g-PVPI microsphere emulsion, the corresponding peak shifted to 3424 cm^−1^, and this shift may be attributed to the presence of iodine. The absorption peak at 1643 cm^−1^ in the spectrum of CMC can be assigned to the C = O stretching vibration, and an analogous peak appears at 1601 cm^−1^ in the spectrum of the CMC-g-PVPI microspheres. Overall, these analyses indicate the successful graft polymerization of CMC and PVPI.

To evaluate the antibacterial property of CMC-g-PVPI microspheres, we cultured *S. aureus* with or without 2% CMC-g-PVPI microspheres solution at 37°C for 24 h. Figure S1 shows that the growth of *S. aureus* could be inhibited by CMC-g-PVPI microspheres while there was no inhibition in the control group. Therefore, CMC-g-PVPI microspheres can prevent bacterial infections in wound healing.

### 3.2. Application of CMC-g-PVPI microspheres and CMC-g-PVPI-γ-PGA to diabetic wound healing.

**Figure 3.**
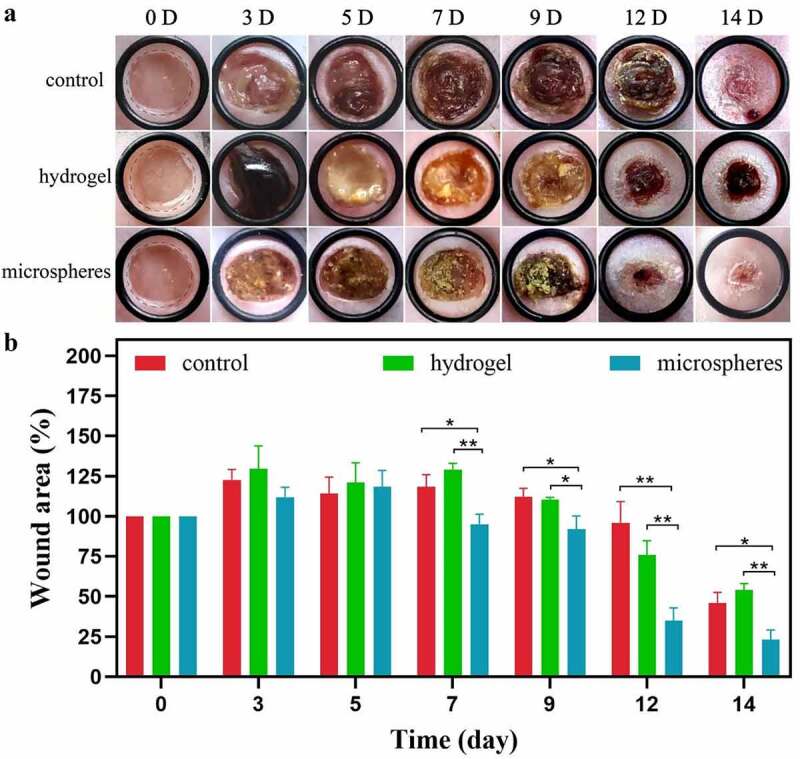
Application of CMC-g-PVPI microspheres and CMC-g-PVPI-γ-PGA to diabetic wounds. (a) Representative photographs of different healing statuses of diabetic wounds treated with CMC-g-PVPI microspheres and CMC-g-PVPI-γ-PGA or untreated (control) on Days 0, 3, 5, 7, 9, 12, and 14. (b) Wound area ratios of all three groups. N = 5, * P < 0.05, ** P < 0.01.

To investigate and compare the healing properties of the CMC-g-PVPI products, we established a diabetic wound model and divided the mice into three groups, with 5 mice in each group. An 8 mm diameter wound was created in the back skin of each mouse. Photographs were taken at different healing times, as shown in Figure 3. As Figure 3a shows, differences among the three groups were not significant until Day 12. The wound area in the microsphere group on Day 12 was obviously reduced, and the healing point was almost reached. However, the wound areas in the hydrogel group and the control group was only slightly different from the original wound area. Figure 3b illustrates the calculated wound area ratios. On Day 12, the wound area ratio of the microsphere group was 30%, which was significantly lower than that of the control group (88%). On Day 14, the wounds in the microsphere group were nearly completely healed, and the wound area ratio was 15%, which was much smaller than those of the control group (47%) and the hydrogel group (55%).

**Figure 4.**
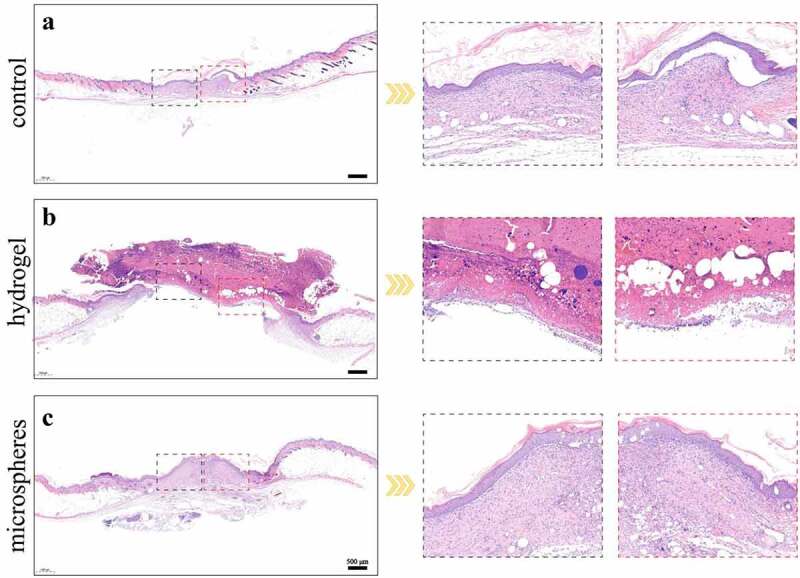
(a-c) H&E staining results of different groups on day 14. Scale bars = 500 µm.

H&E staining images are shown in Figure 4. Significant differences were found between the microsphere group and the other two groups. In the microsphere group, the wounds showed complete and continuous closure (C). In the gel group, as shown in Figure 3, the healing status on Day 14 was the worst among the three groups. The regenerated tissue in this group was not continuous, and the thickness of the epidermis was very shallow at the wound bed. The wounds in the control group exhibited good closure results (A). The repair of the wounds was complete, but when compared with that in the microsphere group, the epidermal layer in the microsphere group was much thicker than that in the control group, demonstrating an accelerated healing effect.

**Figure 5.**
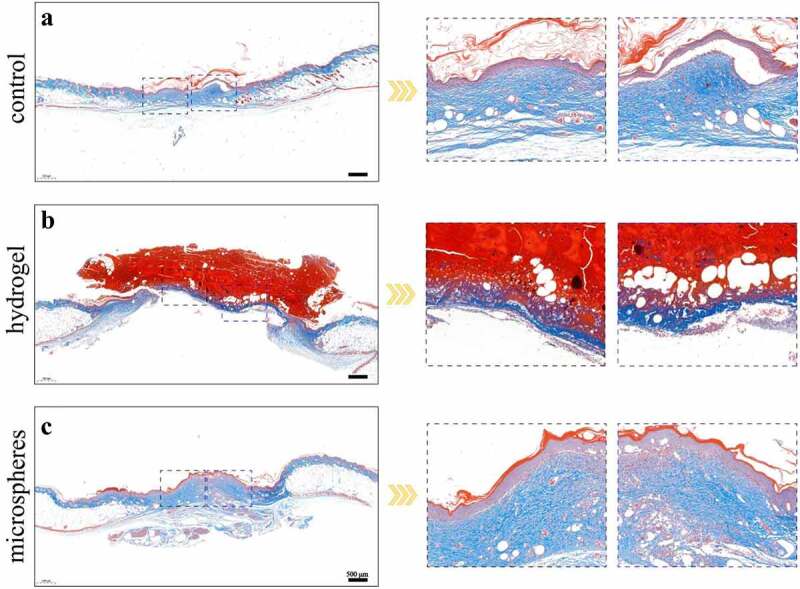
(a-c) Masson trichrome staining results of different groups on day 14. Scale bars = 500 µm.

Figure 5 shows collagen synthesis and deposition in the three groups. The hydrogel group had the least amount of collagen deposition, whereas the control group had good deposition, suggesting a much improved matrix reconstruction. Nevertheless, regenerated collagen tissue was more abundant in the microsphere group than in the control group, indicating a better treatment effect of the CMC-g-PVPI microspheres.

**Figure 6.**
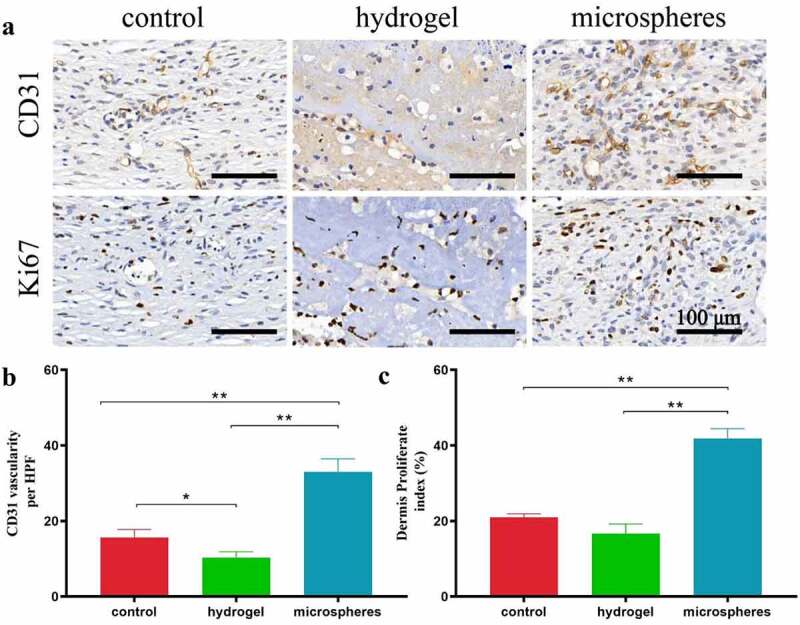
Immunohistochemical staining results of different groups on day 14. Scale bars = 100 µm. (a) Anti-CD31 immunohistochemical staining images of wounds in the control group, hydrogel group and powder group. (b) Anti-Ki67 immunohistochemical staining images of wounds in the control group, hydrogel group and powder group. (c) Quantitative evaluation of anti-CD31 immunohistochemical staining of different groups on Day 14. (d) Quantitative evaluation of anti-Ki67 immunohistochemical staining of different groups on Day 14.

Neovascularization and angiogenesis were also assessed via immunohistochemical assays. Figure 6a shows immunohistochemical staining of the CD31-positive vessels, indicating the capillary density of the wound bed. Compared to the control and hydrogel groups, the microsphere group showed the highest expression of CD31, indicating an accelerated angiogenesis effect of the CMC-g-PVPI microspheres. Ki67 expression is shown in Figure 6b, illustrating cell proliferation activity in the wounds. The microsphere group exhibited 42% dermis proliferation rates, much higher than those of the hydrogel group (15%) and control group (21%), proving the facilitating effect of CMC-g-PVPI microspheres in promoting wound healing.

## 4. Discussion

Chitosan has been reported to promote the expression of TGF-β1 and the production of collagen, stimulating tissue regeneration [26]. In Figure 3, the results of the wound healing test proved the accelerated healing effect of CMC-g-PVPI microspheres. In terms of the four continuous phases of wound healing, namely, hemostasis, inflammation, proliferation, and remodeling [27], scars are formed in the proliferation stage (Day 2 through 14) and remodeling stage (from Day 8) [27,28]. In this study, the wounds in the microsphere group formed a complete scar epithelium between Day 3 and Day 5, whereas in the control group, the completed scar formed on Day 7, indicating better epithelialization and collagen deposition in the microsphere group. From Day 12 to Day 14, the diabetic wounds were in the remodeling phase [28]. Scar shedding occurs in this stage. Marked wound contraction was observed on Day 12. In the control group, the scar formation stage was longer than those of the other two groups, and scars were shed on Day 13. Hydrogels are among the most competitive candidates for wound dressing because of their biocompatibility, oxygen permeability and water retention [27,29]. Many studies have demonstrated the superiority of hydrogel dressings in wound healing [27,30,31]. However, in this study, the microsphere group presented a better wound closure performance than the hydrogel group. The microspheres exhibited good water absorption capacity and could absorb exudate rapidly (Figure 2) [16,29]. Compared to the hydrogel, the microspheres were more effectively fixed on the wound surface, whereas the hydrogel was translocated when the mice moved around. In addition, acetic acid was used to regulate the pH in the process of CMC-g-PVPI-γ-PGA preparation, and the acidic environment may have exacerbated skin irritation and delayed the healing process in the hydrogel group.

To date, chitosan-based materials have been widely applied in wound healing [32], but most of them are in hydrogel form [30,33]. In the present study, we fabricated a complex consisting of carboxymethyl chitosan and PVPI in microsphere form. The microspheres had an appropriate average size (74.89 ± 17.19 µm) (figure 2f), largely decreasing the possibility of migration into the systematic circulation and thus potentially avoiding distal embolus formation [16]. The microsphere form endowed the complex with a larger surface area [34], contributing to the increased exudate contact and absorption [35]. Moreover, compared to hydrogel dressings, microspheres are easier to store and transport, and the administration method of spraying is much more convenient. Wound healing therapies such as stem cell products [36,37] and bioadhesive composites [16] have been widely studied in recent years; however, most of the products used lack antibacterial activity. Among wound dressings with antibacterial properties, antimicrobial drugs and inorganic metal nanoparticles, such as silver (Ag) [38], zinc [39] and iron [40], are mostly used to exert antibacterial effects. However, potential risks of biotoxicity and long-term retention of metals still exist [27]. Herein, we used PVPI for antibacterial activity and to promote the general effect of the copolymer. PVPI avoided the instability and irritation of iodine while exhibiting efficient bactericidal activity, thus providing a relatively stable and sterile environment for healing [20,41].

One of the highlighted strengths of this research is the combination of wound healing materials and bacteriostatic drugs. These two materials are both essential for wound care and are typically applied in sequential order. The complex fabricated by chemical methods integrated the healing and antibacterial effects and greatly reduced the number of procedures and the time required for patients, thus alleviating the high burden on both the family and the health care system. Another strength is the selection of chitosan as the inner skeleton. As chitosan has a long history of being used as a starting material for wound healing, its acceleration of hemostatic activities and cell proliferation provide a foundation for the healing effect. The microsphere form also plays a crucial role in the healing process, with a larger surface area to better absorb exudate.

Limitations also exist in this study. We did not include a chitosan group as one of the control groups to compare the healing properties of the CMC-g-PVPI microspheres with those of CMC, which may have allowed better evaluation of the effect of the complex microspheres. Further studies could be designed to obtain more precise conclusions.

## 5. Conclusion

In summary, a complex of CMC and PVPI was prepared in this study and used on the surface of diabetic wounds. It was observed that the CMC-g-PVPI microspheres had the capacity to accelerate the wound closure process. The advantage of CMC-g-PVPI microspheres is the combination of healing and antibacterial effects, resulting in a faster and novel method for wound repair.

## Supplementary Material

Supplemental Material

## Data Availability

All data and materials during the current study are available from the corresponding author upon reasonable request.

## Abbreviations

IDF, International Diabetes Federation; CMC, Carboxymethyl chitosan; PVPI, polyvinylpyrrolidone-iodine; γ-PGA, γ-poly glutamic acid; CMC-g-PVPI-γ-PGA, [carboxymethyl chitosan-grafted polyvinylpyrrolidone-iodine]-[γ-PGA] hydrogel; CMC-g-PVPI microspheres, carboxymethyl chitosan-grafted polyvinylpyrrolidone-iodine microspheres. SEM, scanning electron microscopy; FTIR, Fourier transform infrared spectroscopy.
