# Supplementary material for: Carboxymethyl chitosan-grafted polyvinylpyrrolidone-iodine microspheres for promoting the healing of chronic wounds
Source: Bioengineered. 2022 Mar 24;13(4):8735–46. doi: 10.1080/21655979.2022.2054911 (PMC9161872; doi:10.1080/21655979.2022.2054911)
Supplement: Supplemental Material [file KBIE_A_2054911_SM5087.docx]

**Supplemental online material**

**Carboxymethyl chitosan-grafted polyvinylpyrrolidone-iodine microspheres for promoting healing of chronic wounds**

Jie Yu^1^, Pei Wang^2^, Mengting Yin^2^, Kaiwen Zhang^1^, Xiansong Wang^2*^ and Bing Han^1*^

1 Department of Endocrinology, Shanghai Ninth People’s Hospital, Shanghai Jiao Tong University School of Medicine, Shanghai, 200011, China

2 Department of Thoracic surgery, Shanghai Key Laboratory of Tissue Engineering, Shanghai Ninth People’s Hospital, Shanghai Jiao Tong University School of Medicine, Shanghai 200011, China

*Corresponding author:

1. mails: hanbing1423@163.com (B. Han); vincentuis@shsmu.edu.cn (X.Wang)

**Supplemental Figure 1.**


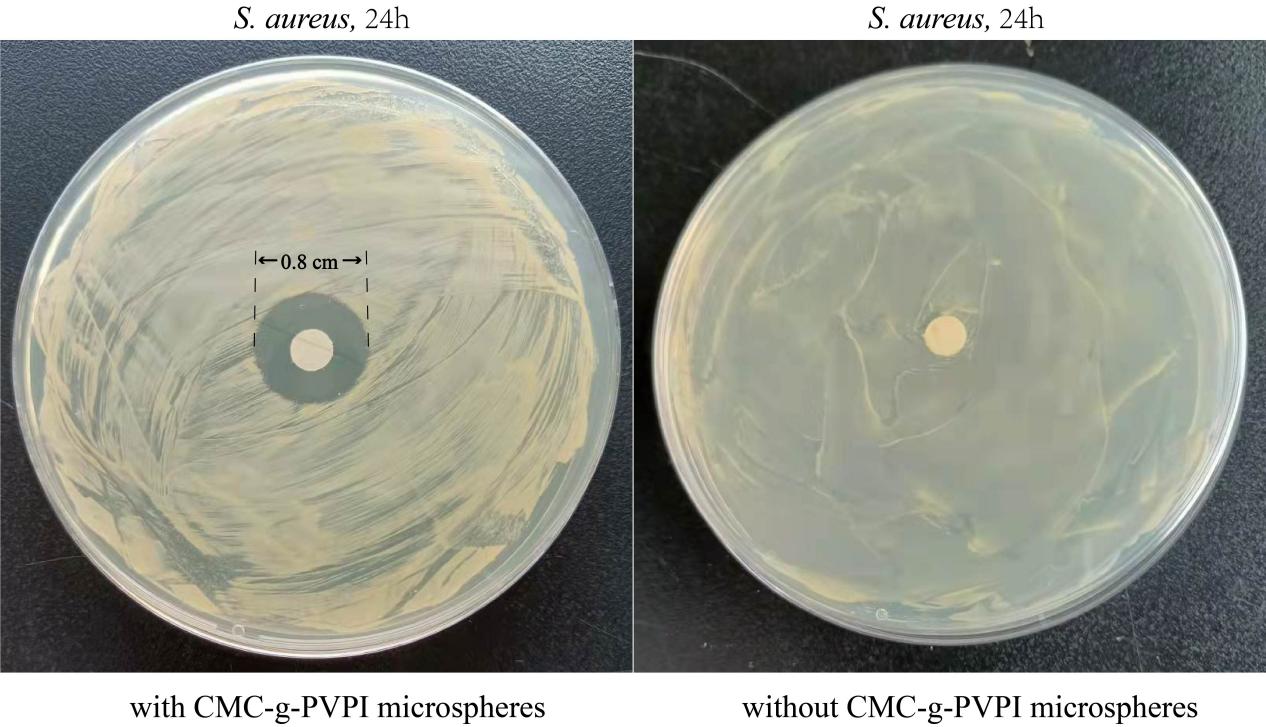


**Figure S1.** Representative photographic images of *S. aureus* bacteria strains treated with or without CMC-g-PVPI microspheres.
